# The role of liver resection for intermediate-stage hepatocellular carcinoma: a meta-regression analysis

**DOI:** 10.1097/JS9.0000000000001034

**Published:** 2024-01-04

**Authors:** Kuo-Chuan Hung, I-Wen Chen, Cheuk-Kwan Sun

**Affiliations:** a Department of Anesthesiology, Chi Mei Medical Center; b Department of Anesthesiology, Chi Mei Medical Center, Liouying, Tainan City; c Department of Emergency Medicine, E-Da Dachang Hospital; d School of Medicine for International Students, College of Medicine, I-Shou University, Kaohsiung City, Taiwan

*Dear Editor,*

We read with great interest the recent article by Bogdanovic *et al*.^1^ titled ‘Liver resection versus transarterial chemoembolisation for the treatment of intermediate hepatocellular carcinoma: a systematic review and meta-analysis’ published in the *International Journal of Surgery*. This issue is important and timely as hepatocellular carcinoma (HCC), a prevalent and often lethal cancer, requires effective management strategies that pose ongoing challenges to clinicians. The comparison between liver resection and transarterial chemoembolization (TACE), two primary treatment modalities, addresses a critical issue on the treatment of intermediate-stage HCC. This study provides vital insights into the improvement of patient outcomes and guidance on clinical decisions in a rapidly evolving field^1^, for which we congratulate the authors on this well-conducted review and meta-analysis.

Although the primary outcome on overall survival showed a significant benefit in favor of liver resection over TACE, there was noticeable heterogeneity (*I* ^2^=79%) between the included studies^1^. This heterogeneity may be attributed to differences in baseline patient characteristics such as age, gender distribution, or tumor burden. In addition, given their inclusion of studies published over a decade (i.e. 2011–2021), the publication year can be a source of heterogeneity in that meta-analysis. Over time, there can be advancements in medical techniques, changes in clinical practice guidelines, improvements in technology, and evolutions in patient care standards. These factors can influence study outcomes and contribute to variability in results across their included studies. Performing meta-regression analyses to assess the impact of such covariables on the effect size would have helped in gaining insights into the issue when heterogeneity is high.

To explore this further, we conducted a meta-regression analysis using the raw data extracted from the original article by Bogdanovic *et al*.^1^ to evaluate the effect of potential moderators, including mean age, proportion of males, mean number of tumors, and year of publication, on the overall survival outcome. Comprehensive Meta-Analysis (Version 4, Biostat, Englewood, New Jersey, USA) was used for analysis as previously reported^2,3^. The meta-regression analyses showed no associations between overall survival and clinical covariables, including age (coefficient: −0.006, *P*=0.834), male proportion (coefficient: −0.018, *P*=0.532) and the number of tumors (coefficient: −0.01, *P*=0.249). However, there was a significant association between publication year and survival outcome (coefficient: −0.08, *P*=0.021) (Fig. 1) with more recent studies showing an increasing benefit of liver resection over TACE. This finding suggests that patient selection, surgical techniques, and/or perioperative care may have been improved in more recent years, enabling better outcomes with liver resection.

**Figure 1:**
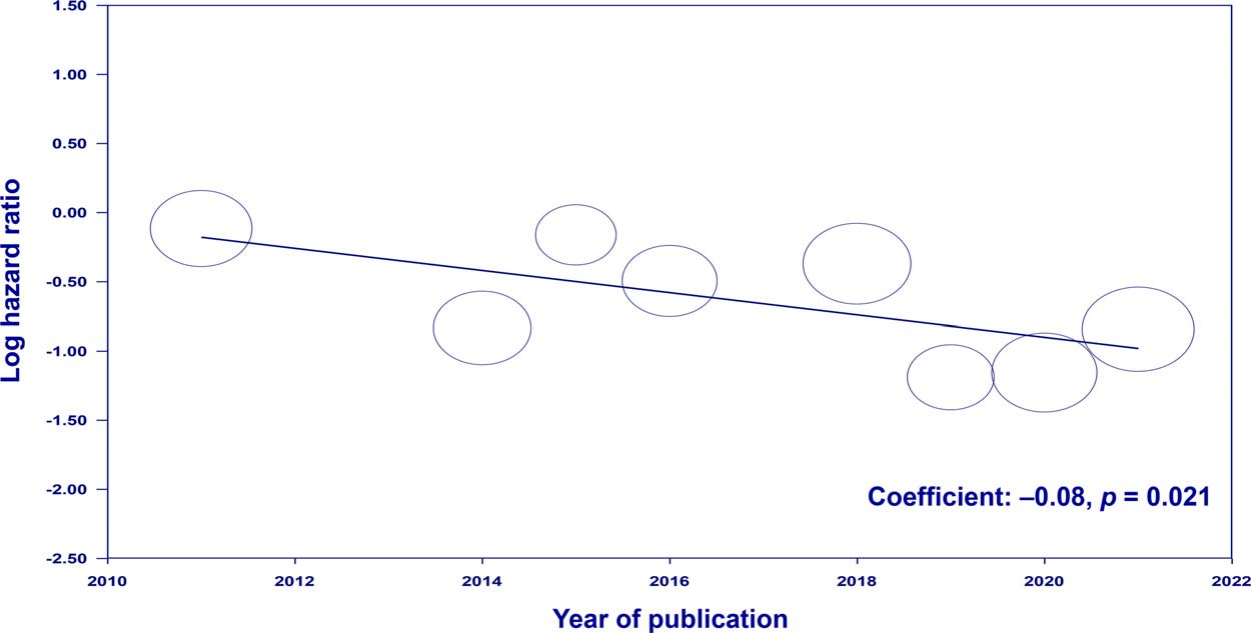
Meta-regression analysis evaluating the correlation between the publication year of studies and the log hazard ratio for overall survival in patients undergoing liver resection versus transarterial chemoembolization for intermediate hepatocellular carcinoma. Each circle represents an individual study; the size correlates with the study’s weight in the analysis. The negative slope indicates a trend towards improved survival with liver resection in more recent studies.

In summary, the well-executed meta-analysis by Bogdanovic *et al*.^1^ offers evidence to support the beneficial role of liver resection in the treatment of intermediate-stage HCC. Our meta-regression analysis provides further insights into factors impacting patient outcomes, highlighting the importance of utilizing modern patient selection criteria and treatment algorithms to identify ideal candidates for surgical management.

## Ethical approval

Not applicable.

## Consent

Not applicable.

## Sources of funding

No external funding was received for this study.

## Author contribution

K.-C.H. and C.-K.S.: wrote the main manuscript text; I-W.C.: prepared Figure 1. All authors read and approved the final version of the manuscript.

## Conflicts of interest disclosure

The authors declare no conflicts of interest.

## Research registration unique identifying number (UIN)

Not applicable.

## Guarantor

Kuo-Chuan Hung.

## Data availability statement

The datasets used and/or analyzed in the current study are available from the corresponding author upon reasonable request.

## Provenance and peer review

This paper was not invited.
